# Attention and P300-based BCI performance in people with amyotrophic lateral sclerosis

**DOI:** 10.3389/fnhum.2013.00732

**Published:** 2013-11-12

**Authors:** Angela Riccio, Luca Simione, Francesca Schettini, Alessia Pizzimenti, Maurizio Inghilleri, Marta Olivetti Belardinelli, Donatella Mattia, Febo Cincotti

**Affiliations:** ^1^Neuroelectrical Imaging and BCI Laboratory, Fondazione Santa LuciaRome, Italy; ^2^Department of Psychology, Sapienza University of RomeRome, Italy; ^3^CNR, Institute of Cognitive Sciences and TechnologiesRome, Italy; ^4^DIAG, Sapienza University of RomeRome, Italy; ^5^Department of Neurology and Psychiatry, Sapienza University of RomeRome, Italy; ^6^Crossing Dialogues AssociationRome, Italy; ^7^Interuniversity Centre for Research on Cognitive Processing in Natural and Artificial Systems (ECONA)Rome, Italy

**Keywords:** brain computer interface, amyotrophic lateral sclerosis, P300, attention, working memory

## Abstract

The purpose of this study was to investigate the support of attentional and memory processes in controlling a P300-based brain-computer interface (BCI) in people with amyotrophic lateral sclerosis (ALS). Eight people with ALS performed two behavioral tasks: (i) a rapid serial visual presentation (RSVP) task, screening the temporal filtering capacity and the speed of the update of the attentive filter, and (ii) a change detection task, screening the memory capacity and the spatial filtering capacity. The participants were also asked to perform a P300-based BCI spelling task. By using correlation and regression analyses, we found that only the temporal filtering capacity in the RSVP task was a predictor of both the P300-based BCI accuracy and of the amplitude of the P300 elicited performing the BCI task. We concluded that the ability to keep the attentional filter active during the selection of a target influences performance in BCI control.

## Introduction

Brain computer interface (BCI) exploits neurophysiological signals to control external devices for a range of applications (Wolpaw and Wolpaw, 2012) such as communication, environmental control, movement control and motor rehabilitation. In the last years the scientific community has made substantial efforts to bring usable BCIs for communication from the laboratory to severely disabled users’ home (Sellers et al., 2010). Amyotrophic lateral sclerosis (ALS) is a neurodegenerative disease causing progressive physical disabilities due to the affection of motor nervous system. This generally leads to death from respiratory failure but life can be extended for several years through artificial ventilation. With the advance of the disease people with ALS may become totally paralyzed and be effectively “locked in” (Oliveira and Pereira, 2009).

Because of the motor characteristics of the disease at the ultimate stage, people with ALS are considered as potential users for the BCI for communication. Studies on BCIs controlled by people with ALS were conducted (Sellers and Donchin, 2006; Hoffmann et al., 2008; Nijboer et al., 2008). The possibility of a long-term independent home use for severely disabled people with ALS was also demonstrated in a single-case report (Sellers et al., 2010). Home based BCI use significantly contributed to quality of life and productivity of the user.

### P300-based BCIs

Among electroencephalographic (EEG) features utilized as input to control a BCI, the Event Related Potentials (ERPs) are transient electric potential shifts time locked to an external event. Such EEG modulations reflect various aspects of cognitive processing. In a typical ERP-based BCI paradigm an adapted oddball task (Fabiani et al., 2007) is implemented. The standard oddball paradigm consists of the presentation of a deviant stimulus in a stream of standard stimuli, the former eliciting the P300 component.

The P300 is a positive deflection typically appearing about 300 ms after the stimulus presentation serving as a link between stimulus characteristics and attention. Two distinct P300 subcomponents have been characterized, the frontal P3a and the central parietal P3b (Polich, 2007). Along with the term P300 we will refer to the P3b component, which has been proposed to reflect memory storage as well as serving as a link between stimulus characteristics and attention (Näätänen, 1990; Patel and Azzam, 2005).

A BCI system which detects occurrences of the P300 in its input EEG signal is usually referred to as P300-based BCI in the literature. Farwell and Donchin (1988) presented the first visual P300-based BCI paradigm (P300 speller) consisting of a 6 by 6 symbol matrix wherein symbols were arranged within rows and columns. Throughout the course of a trial, rows and columns were serially intensified in a random order. The task of the participants was to focus attention on the desired letter which represented the “rare event” of the oddball paradigm and which elicited the P300. The computer identified the attended item as the intersection of the row and column that elicited the largest P300.

Thereafter, many studies were conducted on the P300 speller (Nijboer et al., 2008; Kleih et al., 2010). Other similar visual P300-based devices have also been developed and investigated with able and not able-bodied participants (Piccione et al., 2006; Sellers and Donchin, 2006; Riccio et al., 2011; Zickler et al., 2011).

Only a few studies investigated the relationship between P300-based BCIs and healthy subjects’ physiological and electrophysiological characteristics. Kaufmann et al. (2012) identified a predictor of P300-based BCI performance, with 34 healthy participants, in the resting heart rate variability (HRV), considered as an index of prefrontal inhibition in tasks requiring executive control. In a further study involving a sample of 40 healthy participants, Halder et al. (2013) underlined a correlation between the amplitude of N2 component elicited during an auditory oddball and the performance both at an auditory P300-based BCI and at a visual P300-based BCI. A correlation between amplitude of the late potential (400–600 ms) elicited by the auditory oddball and performance in a visual P300-based BCI was also reported.

Only one study (Mak et al., 2012) investigated the relationship between P300-based BCI and specific features of the EEG in a group of subjects with ALS. Three types of EEG features were identified as predictors of P300-based BCI performance: the root-mean-square amplitude, the negative peak amplitude of the event-related potential to target stimuli and the frequency power in the theta band.

Specifically there is no knowledge about the cognitive capabilities that influence the ability to control a P300-based BCI. Possible candidates of such capabilities could be found in the selective attention and in the visuo-spatial working memory systems.

### P300 relationship with attention and visuo-spatial working memory

Attention and working memory are considered as cognitive processes underlying P300 amplitude.

Johnson’s (1986) “triarchic model” of the P300 amplitude described many experimental variables affecting P300 amplitude with three general factors: *subjective probability*, *stimulus meaning* and *information transmission*.

The *subjective probability* was considered as an externally determined information and the *stimulus meaning* as an internally determined information. Johnson pointed out that the extent to which this two factors operate depends on the *information transmission*, which can be influenced by two independent variables: an external condition, i.e., *the equivocation*, and an internal condition, i.e., *the attention*.

It was suggested that the P300 is a manifestation of a “context updating” activity occurring when one’s model of the environment is revised (Donchin and Coles, 1988). Polich (2003) in the “P300 context-updating model” allocated the process which generates the P300 to an attention driven comparison process by comparing the presented stimulus with the previous event in the working memory. If the stimulus environment is updated, the P300 potential is generated. In a later review, Polich (2007) underlined that selective attention (Kramer et al., 1985) and memory processing (Donchin, 1981) affect P300 amplitude.

Selective attention is defined as the ability to focus our cognitive resources on one relevant aspect of the environment while ignoring irrelevant aspects. The visuo-spatial working memory is defined as the maintenance and or manipulation of task-relevant visuo-spatial information for brief periods of time to guide subsequent behavior.

The concept of attentional filters appeared in literature since the 1950s (Broadbent, 1958). Later, the feature integration theory of attention (Treisman and Gelade, 1980) suggested that we can detect and identify separable features from different objects at an early stage and in parallel, whereas the identification of conjunctions of features (e.g., color and shape) require focal attention to be directed serially to each object location, as a “spotlight”.

One popular way to study selective attentional effects is to require subjects to perform a *visual search task*: participants are presented with a visual display comprising a number of items, and they are asked to manually respond if a predefined target is contained in the display (for a review on selective attention see Quinlan, 2003).

In a typical visual search task, stimuli are spatially selected; a task used to investigate the temporal modality of attentional filtering is the Rapid Serial Visual Presentation (RSVP) task. During the RSVP task, stimuli are presented to the participant in the same location on a screen at a rate of 6–20 items/s (Shapiro et al., 1994). The task is to identify one or more targets embedded in the stream of stimuli. A particular condition was found when participants had to report two targets, with a target presented first in the stream (T1) and a second target (T2) presented immediately after. If both targets have to be attentively processed, detection accuracy for the second target (T2) is usually strongly impaired when T2 is presented between 200 and 400 ms after T1. This failure to accurately report T2 has been termed the attentional blink (AB; Raymond et al., 1992). One of the models explaining the AB effect states that the attentional switching between the two targets in a RSVP trial seems to involve an efficient reconfiguration of the filter which analyzes the incoming stimuli (Di Lollo et al., 2005): the filter is initially configured to process T1 and to exclude the distracter items while the central processor gives temporal control signals to maintain the processing. Upon the arrival of the first target the central processor becomes engaged in stimulus processing and response planning: the second target is thus not processed efficiently until the first target has been fully processed and the central processor has re-established endogenous control over the system’s configuration. Di Lollo et al. (2005) refer to this as a temporary loss of control. Conversely, T1 may also be masked by T2.

Following Bundesen’s (1990) unified theory of visual recognition and attentional selection, perceptual categorizations of elements in the visual field is linked to the limited-capacity of the visual system, i.e., the capacity to filter irrelevant information from a visual scene directly affects the content of the visual working memory. Thus, we can assess the filtering capacity also by using a multi-element array of visual objects presented simultaneously, with a subset of objects indicated as targets and the others as distracters. However, even if the cognitive substrate is similar, as suggested by Bundesen (1990), the attention filtering in a RSVP task operates in a temporal domain whereas in a visuo-spatial working memory task we could assess the capacity to attentionally “filter” distracters in the spatial domain (see Treisman and Gelade, 1980).

A typical visuo-spatial working memory task is the change detection (CD) task, in which participants are presented with an array of one or more items to be remembered after the array is turned off during an interval of seconds. Following this interval a second array is presented and the participant has to judge if it is identical to the first one. Vogel and Machizawa (2004) and Vogel et al. (2005) used this procedure to investigate the electrophysiological basis of visuo-spatial working memory capacity and of the filtering efficiency in controlling access to working memory. Their procedure consisted in two tasks. The first task aimed to investigate the visual memory capacity by presenting an array of 3–4 differently orientated rectangles and requiring the subject to report if, at the second presentation, the rectangles orientation was identical or different from the original. In the second task the subject had to selectively remember only a few relevant items from within an array. The authors found that if the subject was able to efficiently filter the distracters, the electrophysiological substrate modulated by the number of items held in memory (i.e., contralateral delay activity (CDA)) had the same amplitude in the two conditions, with and without distracters (maintaining the same number of relevant items).

Other studies reported that the P300 amplitude was modulated by awareness of the item change in a CD task, with higher amplitude when changes were detected than when changes were not detected (Koivisto and Revonsuo, 2003; Pourtois, 2006).

To investigate the relationship between a range of attentional and working memory processes and the visual P300-based BCI performance, we specifically investigated the temporal dynamics of attention by using a RSVP task (Kranczioch et al., 2007) and the spatial dynamics of attention for visual working memory consolidation by using a CD procedure, as in Vogel et al. (2005).

We hypothesized an association of the parameters reflecting the temporal filtering capacity (RSVP task), the attentive update speed (RSVP task), the memory capacity (CD task) and the spatial filtering capacity (CD task) with the amplitude of the P300 elicited during the P300 speller task and consequently with the P300-based BCI performances.

## Materials and methods

### Participants

We recruited the participants at the ALS center of the Policlinic “Umberto I” of Rome. From a starting pool of volunteers, we excluded from the study the participants who had previous neurological or psychiatric disorders, degenerative diseases other than ALS, any hindrance in the acquisition of EEG data from the scalp (e.g., wounds, dermatitis), severe concomitant pathologies (fever, infections, metabolic disorders, severe heart failure), episodes of reflex epilepsy. To be involved in the study the volunteers had to have at least one preserved communication channel.

Thus, we included in the study a total of nine volunteers, all naïve to BCI training, (three women; mean age = 59.7 ± 12.3) with definite, probable, or probable with laboratory support ALS diagnosis (mean ALSFRS-R scores: 32.4 ± 8.2; Cedarbaum et al., 1999). Due to the fact that one participant did not perform the behavioral tasks, only the data of eight participants out of nine (three women; mean age = 58 ± 12; mean ALSFRS-R scores: 31.8 ± 8.6) were reported in this article (Table 1).

**Table 1:** **Demographic and clinical related data of experimental participants (N = 8)**.

**Participants**	**Age**	**Sex**	**ALSFRS**	**Onset**
1	56	M	13	Spinal
2	59	M	37	Spinal
3	43	M	33	Spinal
4	75	F	38	Bulbar
5	60	F	34	Bulbar
6	40	M	31	Spinal
7	61	M	28	Bulbar
8	72	F	41	Bulbar

The study was approved by the ethic committee of Fondazione Santa Lucia, Rome and all participants provided an informed consent.

### Experimental protocol

The experimental protocol consisted in two sessions performed on two different days. The first was a BCI session: ALS participants were asked to control a 6 by 6 P300 speller. During the second session the participants were involved in the screening of their attentional filtering efficiency and working memory capacity and were asked to perform two behavioral tasks: a RSVP task (Kranczioch et al., 2007) and the CD task (Vogel et al., 2005).

#### P300 speller interface

Scalp EEG signals were recorded (g.MOBILAB, g.tec, Austria) from eight channels according to 10–10 standard (Fz, Cz, Pz, Oz, P3, P4, PO7 and PO8; Chatrian et al., 1985; Krusienski et al., 2006) using active electrodes (g.Ladybird, g.tec, Austria). All channels were referenced to the right earlobe and grounded to the left mastoid. The EEG signal was digitized at 256 Hz. Data acquisition and stimuli delivery were managed by the BCI2000 framework (Schalk et al., 2004).

**Figure 1:**
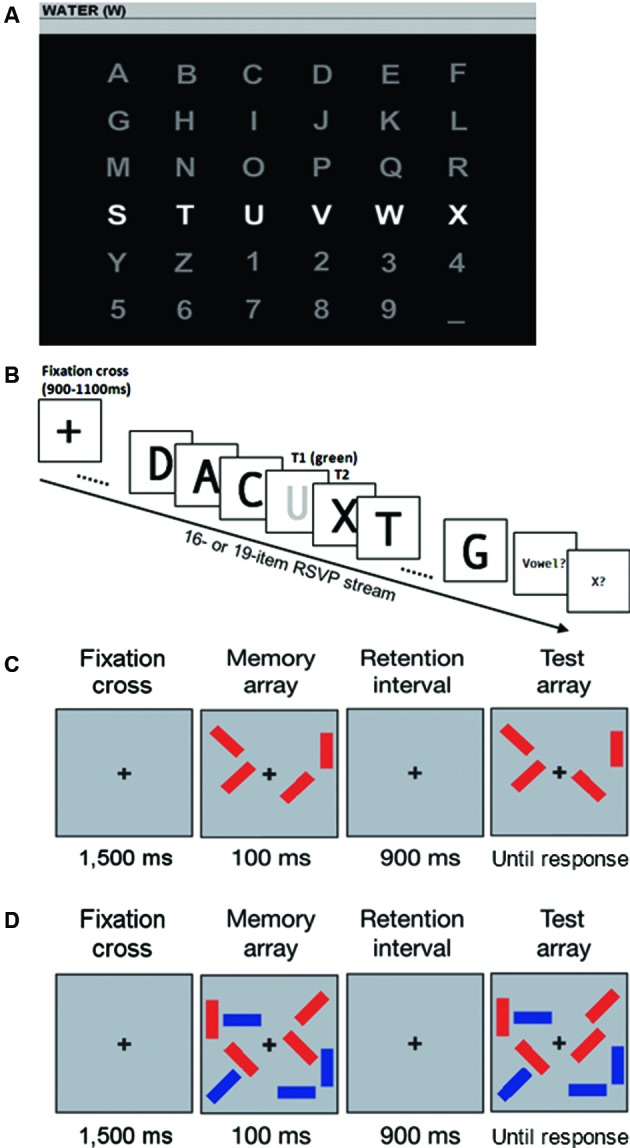
**Tasks presented to participants**. **(A)** P300 speller interface. **(B)** RSVP task: T1 is the letter “U” (presented in green); T2 (X) follows T1 with no intervening distracter (lag 1). **(C)** CD *baseline task*: In the test array, one of the four items changes orientation. **(D)** *Selection task* of the CD task: the participant is asked to focus on the red items ignoring the blue items. In the test array, one of the four red items changes orientation.

Participants were required to copy spell seven predefined words of five characters each (runs), by controlling a P300 speller (Farwell and Donchin, 1988). The latter consisted of a 6 by 6 matrix containing alphanumeric characters (Figure 1A). Rows and columns on the interface were randomly intensified for 125 ms, with an inter stimulus interval (ISI) of 125 ms, yielding a 250 ms lag between the appearance of two stimuli (stimulus onset asynchrony, SOA). For each character selection (trial) all rows and columns were intensified 10 times (stimuli repetitions) thus each single item on the interface was intensified 20 times. Participants were seated facing a 15” computer screen placed at eye level approximately one meter in front of them. The angular distance subtended by the speller was of 15 degrees. A single flash of a letter at the beginning of each trial cued the target to focus. In the first three runs (15 trials in total) EEG data was stored to perform a calibration of the BCI classifier. Thus no feedback was provided to the participant up to this point. A stepwise linear discriminant analysis (SWLDA) was applied to the data from the three calibration runs (i.e., runs 1–3) to determine the classifier weights (i.e., classifier coefficients) (Krusienski et al., 2006). These weights were then applied during the subsequent four testing runs (i.e., testing set; runs 4–7) when participants were provided with feedback. EEG potentials between 0 and 800 ms after each stimulus onset were decimated by replacing each sequence of 12 samples with their mean value and used for the analysis. The next four runs (20 trials in total) characterized a testing phase in which feedback was provided by showing each spelled character. In cases of error the feedback was represented by a dot (instead of the wrongly typed character) to minimize frustration of the participants.

#### Rapid Serial Visual Presentation task

Temporal attention capabilities of participants were assessed by using the RSVP task as in Kranczioch et al. (2007). In the RSVP task (Figure 1B), two targets were embedded in a stream of distracter stimuli. Each stream included 16 or 19 items, of which one or two were targets. All stimuli were presented at central fixation on a monitor with a white background at a presentation rate of 10 Hz. Each letter subtended a region on the screen of about 1.5° × 1.38° of visual angle. Distracters were black capital consonants (except F, K, Q, X, Z) and the distracter sequence was pseudo-randomly extracted, with the constraint that the same letter could not be presented within three sequential positions. The first target (T1) was a green letter, which could either be a vowel or a consonant (except F, K, Q, X, Z), and the second target (T2) was a black capital “X”. Each trial started with the presentation of a fixation cross for 900 to 1100 ms (mean 1000 ms). T1 was presented randomly as 4th, 5th, 6th or 7th item in the stream. In 20% of trials T2 was not presented, whereas it followed with no (lag 1), one (lag 2), three (lag 4) or five (lag 6) intervening distracters, in 20% of trials for each condition. After the end of the stimulus stream, two successive screens appeared asking whether the green letter (T1) was a vowel and whether the black X (T2) was contained in the stimulus stream, as in Kranczioch et al. (2005, 2007). Participants completed 20 practice trials before completing 160 experimental trials, presented in a randomized order (32 trials for each of the five conditions).

Due to the motor disabilities of the participants, they were asked to give a binary response (yes or no) to the operator with the residual communication channel.

#### Change Detection task

Memory capacity and spatial filtering capacity were screened by using a CD procedure. The participants completed two CD tasks (a *baseline* task and a *selection* task). In both tasks, all stimuli were presented on a gray background and each trial started with a fixation cross, presented for 1500 ms at the center of the screen. An array of rectangles (memory array) with varying orientation to memorize was then presented for 100 ms. Each memory array included between three and eight colored rectangles (each subtending a region of about 0.4° × 1.7° visual degrees) presented within a 9.8° × 7.3° region on the screen. Stimulus positions were randomized on each trial, with the constraint that the distance between objects was at least 2° (center to center). Each rectangle was either blue or red and had one of four possible orientations (vertical, horizontal, left 45° and right 45°) randomly chose. The memory array was followed by a retention interval of 900 ms and then by a second array of rectangles (test array). The test array could either be identical to the original memory array or different by, in orientation to one of the previously presented rectangles.

In the *baseline task* (Figure 1C), each memory array consisted of three or four rectangles of the same color (all blue or all red), with one out of four possible orientations (vertical, horizontal and two diagonals) randomly extracted. The participants were asked to report if the orientation of the rectangles in the test array was identical to the ones in the memory array. The task included 10 practice trials and 40 experimental trials for condition, for a total of 80 trials, fully randomized in a unique block.

In the *selection task* (Figure 1D), each memory array consisted of six or eight rectangles. Half of the rectangles were blue and the other half were red. Half of the participants were instructed to memorize the blue rectangles, considered as targets, and to ignore the red rectangles. They were asked thus to report if the orientation of the blue rectangles in the test array was identical to the ones in the memory array. Half of the participants were instructed to memorize the red rectangles, considered as targets, and to ignore the blue ones. In this case they were asked to report if the orientation of the red rectangles in the test array was identical to the ones in the memory array. This task included 10 practice trials and 40 experimental trials for condition, for a total of 80 trials, fully randomized in a unique block.

Due to the motor disabilities of the participants, they were asked to give a binary response (yes or no) to the operator with the residual communication channel.

### Data analysis

#### P300 morphology

**Figure 2:**
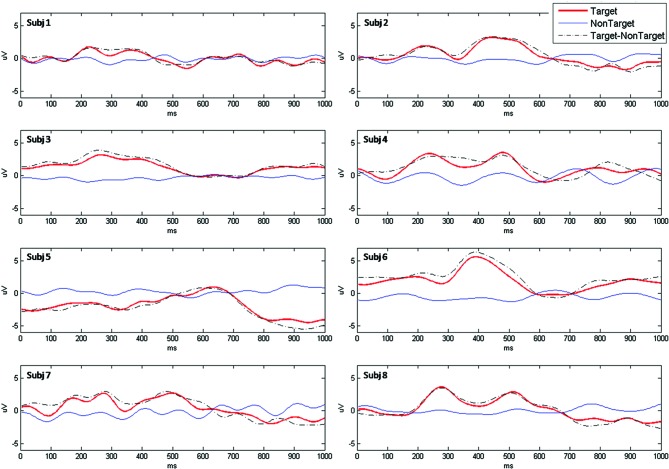
**EEG amplitude as a function of time, between 0 (stimulus onset) and 1000 ms, registered during BCI session, for N = 8 participants**.

EEG data was high pass and low pass filtered with cut off frequencies of 0.1 Hz and 10 Hz respectively using a 4th order Butterworth filter. In addition, a notch filter was used to remove 50 Hz contamination due to the AC interference. Data was divided into 1000 ms long epochs starting with the onset of each stimulus. Epochs in which peak amplitude was higher than 70 μV or lower than −70 μV were identified as artifacts and removed. A baseline correction was done based on the average EEG activity within 200 ms immediately preceding each epoch. The average waveform for both target and non-target epochs was computed for each trial in order to assess P300 peak amplitude. Particularly, amplitude of the P300 potential in Cz was defined as the highest value of the difference between target and non-target average waveforms in the time interval 250–700 ms (P300 amp, Figure 2).

#### Single trial classification

To provide an estimate of the classifier accuracy we considered the binary classification problem target vs. non-target (Blankertz et al., 2011) that takes into account the correct classification of a target or of a non-target. Frequency filtering, data segmentation and artifact rejection were conducted as in P300 morphology section. EEG data were then resampled by replacing each sequence of 12 samples with their mean value, yielding 17 × 8 samples per epoch (eight being the number of channels), which were concatenated in a feature vector (Krusienski et al., 2006). A seven-fold cross-validation was used to evaluate the binary accuracy (BA) of the classifier on each participant’s dataset. For each iteration we applied a SWLDA on the testing dataset (consisting of six words) to extract the 60 most significant control features (Draper and Smith, 1998) and we assessed the BA on the training dataset (the remaining word).

### Behavioral data analysis

The detection accuracy of T1 (T1%) in the RSVP task was considered as an index of the temporal attentional filtering capacity of the participants. Since the detection accuracy of T2 (T2%) was considered as an index of the capability to adequately update the attentive filter, only trials in which T1 had been correctly identified were selected in order to determine T2%.

To investigate the memory capacity we only considered memory arrays with the highest number of rectangles (4 or 8). According to Cowan (2001), we defined the number of items held in memory (K) as K = S(H−F), where S is the size of the array (highest number of item to memorize, S = 4), H is the observed hit rate and F is the false alarm rate. We calculated the K index for the *baseline task* (K_b_) and for the *selection task* (K_s_). To screen for the *attentional spatial filtering capacity* (α) of the participants we subtracted the K_s_ from the K_b_ (α = K_b_ − K_s_).

### Statistical analysis

Statistical analysis was aimed to test the hypothesis that attentional and memory substrates influence the performance in the P300 speller task and the features of the P300 elicited while performing this task.

Since T1%, T2%, BA, and P3 amp were normally distributed, the Pearson’s correlation coefficient of T1%, T2% with the BA and the P3 amp was computed.

Since K_b_ and α violated the assumption of normality, they were correlated with BA and P3 amp by means of the non-parametric Spearman’s correlation test.

For the parameters whose correlation was statistically significant we performed two regression analyses in which attentional parameter (T1%) was considered as the independent variable and BA and P300 amp were considered as dependent variables.

## Results

One participant (participant 8) was excluded from the analysis regarding the RSVP task, due to technical problems encountered during data recording. However, that participant’s data collected during the CD task were considered in the analysis. One participant did not perform the *selection task* of the CD task because of eye fatigability (participant 5); this lack of data did not allow us to calculate and consider in the analysis her *attentional spatial filtering capacity* (α). One participant (participant 2) refused to perform both the CD tasks (*baseline task* and *selection task*) due to fatigue.

In brief, analysis on the scores collected by means of the RSVP task (T1% and T2%) were performed on seven participants. Analysis on the scores collected by means of the CD task (K_b_ and K_a_), were performed on seven participants when considering the K_b_ scores, and six participants when considering the K_a_ scores.

Mean online accuracy in performing the BCI task was 97.5% (SD = 3.8, range = 90–100%, N = 8; Table 2), the offline BA was on average of 87.4% (SD = 2.4%, range = 84.5–92.3%, N = 8; Table 2). The mean amplitude for P300 amp in Cz, was 3.3 μV (SD = 1.6, range = 1.1–6.5 μV, N = 8).

**Table 2:** **Participants’ scores with means and Standard Deviations (SD)**.

**Participants**	**T1%**	**T2%**	**P300 amp**	**K_b_**	***α***	**BA%**	**Online acc %**
**1**	69.4	60.8	1.90	3.2	0.2	84.5	95
**2**	73.7	77.3	2.45	–	–	86.3	100
**3**	73.7	77.9	4.40	3.2	1.4	87.2	90
**4**	77.5	50.4	3.45	1.0	1.0	85.9	100
**5**	65.0	51.3	1.09	2.2	–	86.2	95
**6**	96.2	87.1	6.55	3.2	0.0	88.6	100
**7**	85.0	69.3	3.26	1.6	0.4	88.6	100
**8**	–	–	3.18	1.6	1.0	92.3	100
**Mean ± SD**	77.2 ± 10.4	67.7 ± 14.1	3.3 ± 1.6	2.3 ± 0.9	0.6 ± 0.5	87.4 ± 2.4	97.5 ± 3.8

*T1%: accuracy in detection of T1 in the RSVP task; T2%: accuracy in detecting T2, if T1 is correctly detected, in RSVP task; P300 amp: amplitude of the difference between the P300 elicited by the target and the P300 elicited by the no-target in Cz (P300-speller task);* K_b_: K *index (number of items held in memory) for the baseline task;* α: *spatial filtering capacity (*K_b_−K_s_*);* BA%*: offline* BA*; Online acc %: online accuracy in P300-speller task*

In the RSVP task, mean accuracy of detection for T1 was 77.2% (SD = 10.4%, range = 65–96.25%, N = 7) and for T2 67.7% (SD = 14.1%, range = 50.3–87.1%, N = 7).

A significant positive correlation was observed between T1% and the offline BA (*r* = 0.79, *p* < 0.05), showing that participants with higher T1% had a higher accuracy in the offline binary classification. To estimate the predictive value of T1% on the BA we computed a regression analysis which resulted in an *F* = 8.34 with a *p* < 0.05, indicating that the variance of the binary performance is predictable by the participant temporal filtering capacity, with *β* = 0.79.

A significant positive correlation was found between T1% and P300 amp in Cz (in *r* = 0.84, *p* < 0.05) showing that participants with higher T1% had a larger P300 amp in Cz. As a result of the linear regression, T1 accuracy was significantly predictive of P300 amp in Cz (*F* = 16,23 with a *p* < 0.05) with *β* = 0.87.

No significant correlation was found between T2%, the offline binary performance and P300 amp in Cz.

The number of items held in memory by the participants (K_b_) in the CD task was on average 2.3 items (SD = 0.9, range = 1–3.2, N = 7). The α value was on average 0.6 (SD = 0.5, range = 0–1, N = 7).

No significant correlation occurred between *K_b_*, α and the two BCI variables (BA and P300 amp).

## Discussion

The purpose of this study was to investigate whether attentive and memory capacities may influence and predict performances in controlling a visual P300-based BCI in a group of people with ALS. We hypothesized that the capabilities of maintaining temporal attentional filters and creating spatial attentional filters would be important cognitive substrates supporting the skill required to operate a visual P300-based BCI.

We found that T1% (RSVP task) was a predictor of the amplitude difference between the P300 elicited by target and the P300 elicited by the non-target in the BCI task. T1% was also found to be a predictor of the offline BA obtained in the BCI task. We considered T1% an index of the capacity of detecting and reporting a target within a stream of distracter stimuli.

We did not find any association between the detection of T2% (RSVP task) and the BCI related variables. No association was highlighted between BCI variables and participants’ memory capacity and spatial attention filtering capacity.

### RSVP task and BCI task

 The detection rate of T1 in the RSVP task can be interpreted as an index of selective attention: it represents indeed the capacity to detect a target within a stream of stimuli, to create a memory trace and to retain it. More precisely it represents the basic capability to temporally filter the target by distracters and to maintain the filter in a range of time, keeping a continuous top-down control (Di Lollo et al., 2005). Such capability varies from subject to subject and we demonstrated that it influences the performances in the BCI task, supporting our hypothesis about the existence of a common cognitive substrate between the two investigated tasks.

As the accuracy of detecting T2 is an index of the speed of attentive update, the missed correlation with the considered BCI variables leads us to speculate that the capacity of dynamically update the attentive filter is less likely to be a cognitive substrate supporting the BCI control. This might be explainable by the nature of P300 speller stimulation: the letter stimuli are statically presented on the screen and the central processor does not need to update the filtering map in order to process the successive stimuli. In the RSVP task indeed, a filtering map is configured for the processing of the T1, a green letter, and has to be dynamically reallocated in order to process the T2. Such dynamic reallocation is not a cognitive substrate supporting the target stimuli processing during the P300 speller task. Thus, an association between the T2% and the accuracy in a BCI task could possibly arise by using a speller in which the pattern of the letters is randomized every three to five sequences, stressing the capacity of participants to reallocate their attentional filter.

Although the sample of participants of the presented study had relatively high-motor functioning, the accuracy of detection of T1 (77.6%) in the RSVP task was lower compared with the scores reported in the RSVP literature. Kranczioch et al. (2005), exploiting the same RSVP paradigm utilized in this study, reported accuracy in T1 detection always above 96.5%. It can be argued that the sample involved in Kranczioch et al. (2005) study, was younger (age 19–34) than the sample involved in our study (age 40–72). Interestingly Georgiou-Karistianis et al. (2007) performed a study about the progressive age-related changing in RSVP task performance (sample ranging in age from 18–82; mean age = 42.51 ± 19.21), showing that age was not associated with a reduced ability to detect T1. The accuracy of T1 detection was indeed of 92% on average. In conclusion we can speculate that the capability to temporally filter a target and to maintain it is weaker in our sample of ALS participants in comparison with the healthy samples reported in others studies investigating performances in RSVP task.

### CD task and BCI task

We hypothesized that the memory capacity and the spatial filtering capacity were associated with the amplitude of the P300 elicited during a P300 speller task and the capability of the participants to control the P300 speller. The lack of relationship between BCI parameters and the variables measured with the CD task did not confirm this hypothesis. According to the Bundesen’s (1990) unified theory of visual recognition and attentional selection, the selection of stimuli to be stored in memory is spatial and feature based. We can speculate that the allocation of attention on the selected item during the BCI task is not based on spatial (being the location of the target letter static) or feature characteristics but on symbolic aspects (e.g., semantic aspects of the target letter).

## Conclusions

We can conclude that the cognitive process which influences the performance in BCI control is not the capability to create an attentive map itself, but to keep it active. Following the Huang and Pashler’s (2007) Boolean Map Theory of visual attention, the attentional filter is achieved by a boolean map, a mechanism of visual access (spatial filter) that divides the visual field into selected and unselected subfields. A top down mechanism creates and maintains over time the Boolean maps. Such top-down mechanism is crucial to control a BCI speller task, allowing the user to set up and maintain the proper attentional map throughout a trial and thus to select the desired letter.

The data reported in the present paper partly clarify the cognitive substrate related to BCI control in people with ALS. This issue could allow future speculations on the factors underlying BCI control failure observed in potential user groups. The awareness about the processes and the clinical features of BCI potential end-users influencing the BCI performance and use, would allow developing flexible systems, adaptable to different clinical profiles. As also suggested by Schreuder et al. (2013), thus we consider crucial to adapt BCI-based devices to end users with a range of cognitive profiles.

## Conflict of interest statement

The authors declare that the research was conducted in the absence of any commercial or financial relationships that could be construed as a potential conflict of interest.
